# Assessment of coronary wall thickening in autosomal dominant hyper- immunoglobin E syndrome (AD-HIES) using TRAPD-MRI

**DOI:** 10.1186/1532-429X-18-S1-O5

**Published:** 2016-01-27

**Authors:** Khaled Z Abd-Elmoniem, Nadine Ramos, Saami Yazdani, Steven Holland, Alexandra Freeman, Ahmed M Gharib

**Affiliations:** 1 NIDDK, NIH, Bethesda, MD USA; 2 NIAID, NIH, Bethesda, MD USA; 3 University of South Alabama, Mobile, AL USA

## Background

Autosomal dominant hyper-IgE (AD-HIES), also called Job's syndrome, is a rare primary immunodeficiency caused by dominant mutations in STAT3. AD-HIES is characterized by elevated levels of IgE, an ineffective immune response to several infectious agents, and connective tissue and arterial abnormalities. To date, coronary artery evaluation in AD-HIES patients has been limited to lumenography by CTA or MRA. Direct *in vivo* coronary vessel wall imaging may allow for earlier detection of coronary artery disease, possibly at the subclinical stage, and may lead to more accurate assessment of treatment efficacy.

The goal of this prospective study was to evaluate the coronary artery walls of AD-HIES patients using MRI and compare to healthy subjects and subjects with known coronary artery disease.

## Methods: study population

A total of 28 subjects were included in the study; 10 subjects with AD-HIES, 8 healthy subjects, and 10 patients with with known CAD as proven by coronary Computed Tomography Angiography (CTA). Groups were age- and BMI-matched as shown in the table.

## Coronary wall imaging

free-breathing time-resolved dark-blood (trapd-mri) proximal coronary vessel wall datasets were acquired with a fixed inversion time (TI = 200 ms) and phase-sensitive reconstruction. Data were acquired using a segmented k-space spiral acquisition with spectral spatial excitation, using a 32-channel phased array cardiac received coil and VCG triggering. Images were anonymized, wall thickness was measured as previously published.

## Computer tomography angiography

Multidetector computerized tomography (MDCT) scans with ECG gating were performed in 10 CAD subjects. The MDCT protocol was similar to previously described techniques. Image analysis and interpretation of the axial and the multiplanar re-formatted images were performed using a commercial three-dimensional software tool.

## Results

**Figure 1:**
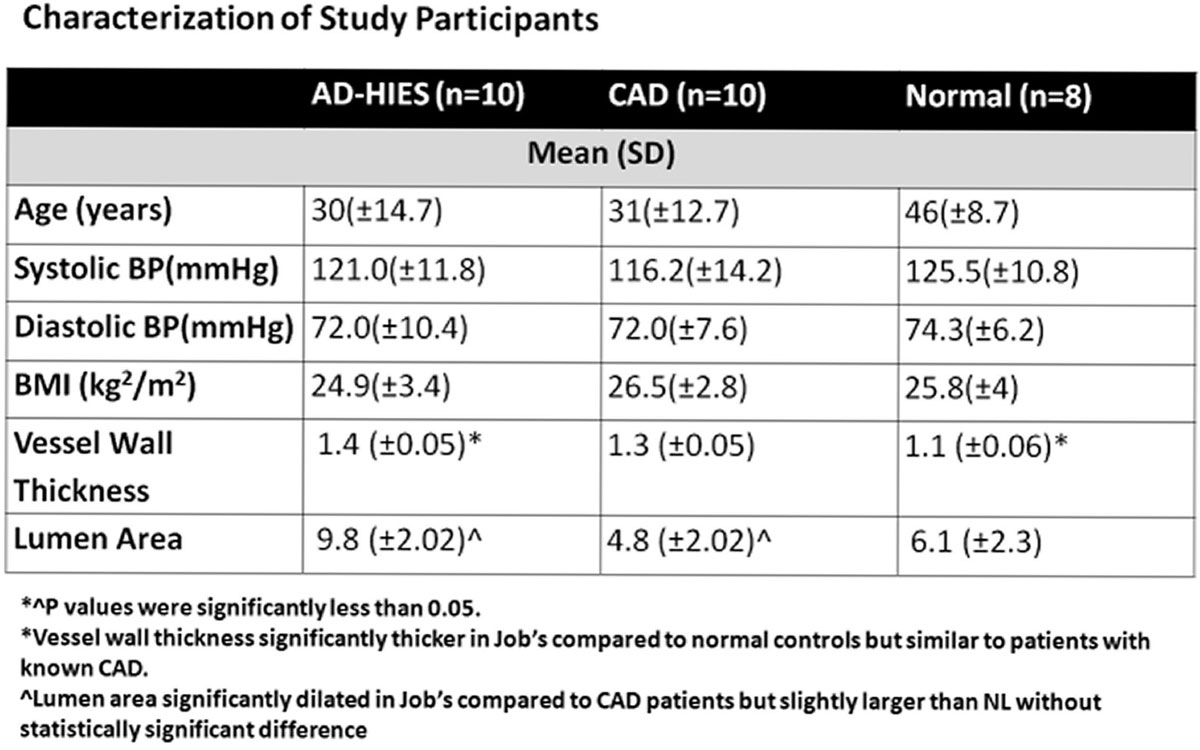
Figure 1

Examples of the coronary vessel wall images in the three groups are shown in Figure 1a. MRI imaging of coronary vessel walls of AD-HIES patients showed thicker vessel walls than those of healthy controls (Table 1). There was no statistically significant difference in vessel wall thickness between non-AD-HIES subjects with atherosclerosis and AD-HIES patients (Figure 1b).

**Figure 2:**
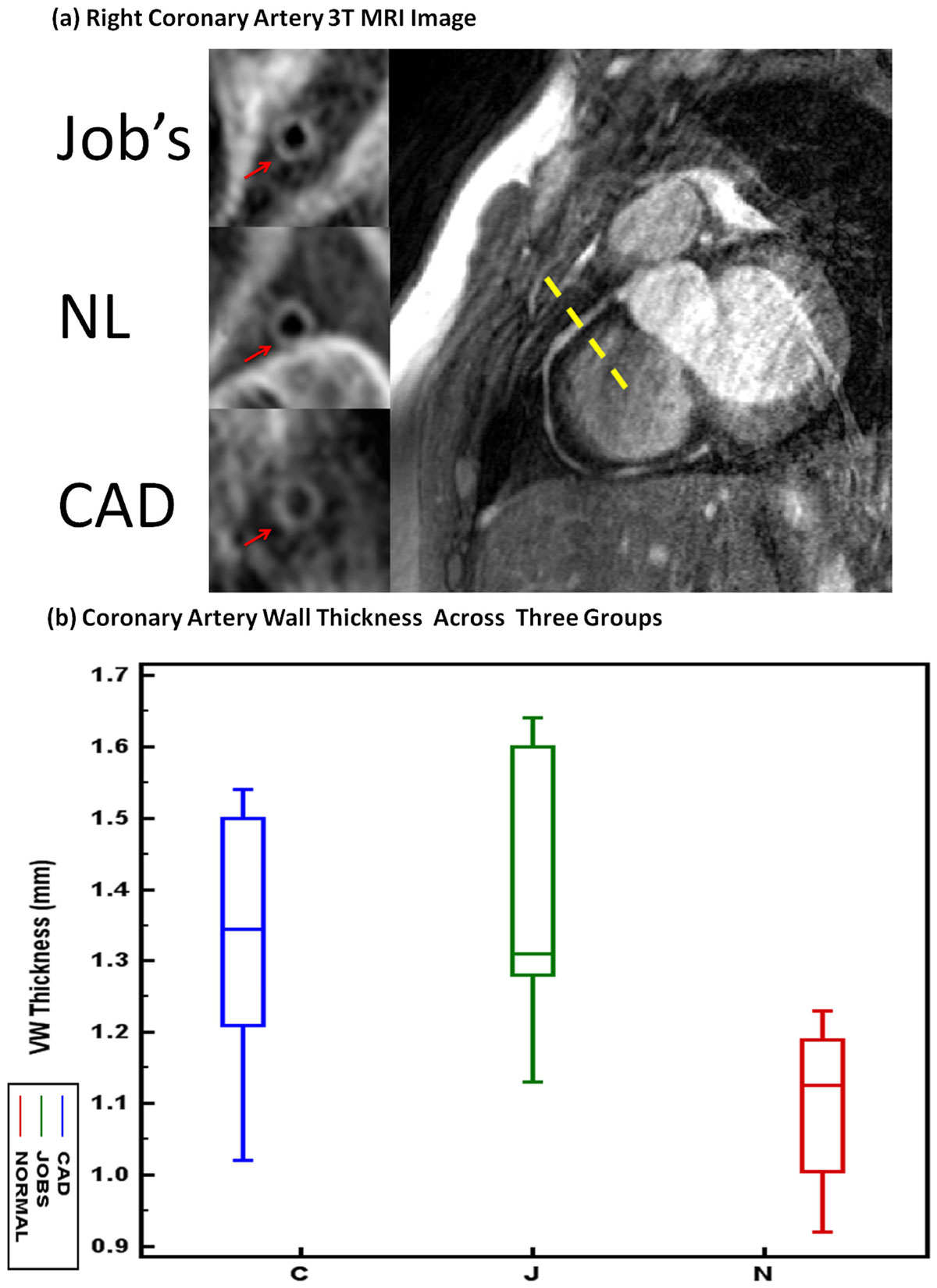
Figure 2

## Conclusions

This is the first study to image the coronary vessel wall of patients with AD-HIES by MRI. The MR vessel thickness of AD-HIES patients was compared to both healthy subjects and patients with known coronary artery disease (CAD) as demonstrated by CTA. The findings demonstrate that the coronary vessel wall in Job's syndrome subjects is thicker than healthy subjects, however, comparable to patients with known CAD. These findings suggest that coronary arteries in Job's syndrome are affected with atherosclerosis, contrary to prior beliefs and study findings.

## References

[1] Abd-Elmoniem KZ (2012). Coronary vessel wall 3-T MR imaging with time-resolved acquisition of phase-sensitive dual inversion-recovery (TRAPD) technique. Radiology.

